# Chemical Profile and Biological Activities of Essential Oil from *Artemisia vulgaris* L. Cultivated in Brazil

**DOI:** 10.3390/ph12020049

**Published:** 2019-04-01

**Authors:** Sonia Malik, Ludmilla Santos Silva de Mesquita, Carolina Rocha Silva, José Wilson Carvalho de Mesquita, Emmeline de Sá Rocha, Jayakumar Bose, Rambod Abiri, Patricia de Maria Silva Figueiredo, Livio M. Costa-Júnior

**Affiliations:** 1 Graduate Program in Health Sciences, Biological and Health Sciences Center, Federal University of Maranhão, São Luís 65085-580, MA, Brazil; 2 Australian Research Council Centre of Excellence in Plant Energy Biology, School of Agriculture, Food and Wine, Waite Research Institute, University of Adelaide, PMB 1, Glen Osmond, SA 5064, Australia; jayakumar.bose@adelaide.edu.au; 3 Department of Pharmacy, Biological and Health Sciences Center, Federal University of Maranhão, São Luís 65085-580, MA, Brazil; ludmilla.ssm@gmail.com (L.S.S.d.M.); biojwill@hotmail.com (J.W.C.d.M.); emmelinesa@gmail.com (E.d.S.R.); figueiredo.patricia@gmail.com (P.d.M.S.F.); 4 Department of Pathology, Biological and Health Sciences Center, Federal University of Maranhão, São Luís 65085-580, MA, Brazil; carolinars@live.com (C.R.S.); livioslz@yahoo.com (L.M.C.-J.); 5 Department of Biochemistry, Faculty of Biotechnology and Biomolecular Sciences, University Putra Malaysia, UPM Serdang 43400, Selangor DE, Malaysia; rambod_abiri@yahoo.com; 6 Young Researchers and Elite Club, Islamic Azad University, Kermanshah, Iran

**Keywords:** anthelmintic, antimicrobial, (compositae), caryophyllene, germacrene-D, humulene, GCMS-chromatography

## Abstract

Essential oil from the leaves of *Artemisia vulgaris* L. (Compositae) cultivated in Brazil was investigated for its chemical composition and biological activities including antibacterial, antifungal, and anthelmintic. The constituents of essential oils isolated by hydro-distillation were examined by GC-MS and a total of 18 components were identified. The essential oil was dominated by oxygenated sesquiterpenes (44.4%), sesquiterpene hydrocarbons (33.3%), and oxygenated monoterpenes (16.6%). Caryophyllene (37.45%), germacrene D (16.17%), and humulene (13.66%) were the major components. The essential oils from *A. vulgaris* showed bactericidal and fungicidal properties against *Staphylococcus aureus* and *Candida albicans*, respectively. Anthelmintic activity against *Haemonchus contortus* was absent in this essential oil. Altogether above results indicate that essential oils from *A. vulgaris* can be used for various medicinal purposes.

## 1. Introduction

Increasing resistance and other side effects caused by the repeated use of similar antibiotics or anti-parasitics enforced the researchers to find suitable natural compounds as alternatives. A large number of plant derived compounds play an important role in the natural defence system against all living microorganisms [1]. In vitro investigations of antimicrobial and antifungal activities have been carried out for several medicinal crops including *Baccharis trimera* [2], *Zingiber officinale* [3,4], *Mikania glomerata* [5,6], *Mentha piperita* [3,7], *Syzygium aromaticum* [8], *Cymbopogon citratus* [9,10], *Allium sativum* [11,12], and *Psidium guajava* [6,11,13]. There is a huge interest in medicinal crops to extract oils and bioactive compounds to use them as food additives to delay or prevent growth and development of microorganisms [14,15,16,17,18].

The genus *Artemisia* is among the most widely distributed and largest genera of family Asteraceae, containing around 500 taxa [19,20,21]. Many *Artemisia* species grow in Northern Africa, North and Central America, and Eurasia [22]. *Artemisia vulgaris* Linn., commonly known as mugwort, is a rhizomatous perennial medicinal plant [23] and is widely used to treat to dyspepsia, rheumatic pains, fevers, diarrhea, worm infestations, vomiting, constipation, cramps, colic, hysteria, flatulence, menstrual problems, distention, epilepsy, to promote circulation, and as a sedative [24,25,26,27].

Several essential oils of aromatic plant are used for their antispasmodic, carminative, anti-parasitical, anti-inflammatory, antimicrobial, anti-helminthic, and insecticidal properties [22]. Biological activities of these essential oils are mainly attributed to volatile compounds, such as α-pinene, camphor, caryophyllene, camphene, germacrene D, 1,8-cineole, and α-thujone [22]. In this respect, various mugwort genotypes growing in different geographic regions showed varied components fraction. For example, oils from Italian mugwort was rich in camphor alone or together with myrcene, 1,8–cineole, or borneol [28,29]; German mugwort contained sabinene, myrcene, and 1,8-cineole [22]; Indian mugwort was rich in camphor, α-thujone, or thujone isomer [30]; French mugwort was rich in camphor, 1,8-cineole, and terpinen-4-ol [31]; Moroccan mugwort had camphor, isothujone, and thujone as major components [32]; Iranian mugwort dominated with α-pinene, menthol, β-eudesmol, and spathulenol [33], Cuban mugwort was rich in sesquiterpene [34]. However, there is no report on the chemical composition of Brazilian mugwort oils.

Knowing the exact chemical composition of Brazilian mugwort oil is critical to identify its biological properties (e.g., antimicrobial, anti-parasitical, and insecticidal). Among all the pathogens, *Staphylococcus aureus* [7], *Escherichia coli* [35], and *Candida albicans* are of great importance to public health [36]. *S. aureus*, a Gram-positive and round-shaped bacterium [7] is known to cause skin and severe bloodstream infections in patients using catheters and has also been frequently associated with pneumonias associated with mechanical ventilation [24]. *C. albicans* is the main etiological agent of candidiasis, the most common pathogen in humans which colonizes the genitourinary tracts, gastrointestinal tracts, teeth, mouth, as well as skin of more than 70% of the healthy population [37,38]. *E. coli* is a bacterium commonly found in the digestive tract of humans and warm-blooded animals. Some strains of *E. coli* can cause serious food-borne diseases. *Haemonchus contortus*, a parasitic gastrointestinal nematode, is a serious threat to small ruminants’ health. Diarrhea, low packed cell volume (PCV), anemia, peripheral, internal fluid accumulation, and dehydration are common signs of this nematode [39]. To ascertain the antimicrobial and anti-parasitical activity of Brazilian mugwort oils, it is necessary to test the efficacy of Brazilian mugwort oils against above pathogens.

Considering aforementioned literature, the main purpose of this study was to assess the chemical composition of essential oil extracted from the leaves of *A. vulgaris* grown in Brazil, and to identify its biological properties.

## 2. Material and Methods

### 2.1. Plant Material and Botanical Identification

Aerial parts (before the onset of flowering) of *A. vulgaris* were collected from plants growing in botanical garden at the Federal University of Maranhão, Sao Luís, Maranhão, Brazil in August 2016. Plants were identified and authenticated by Dr. Eduardo B. de Almeida Junior. The voucher specimens (number 8.637) were kept at the Herbarium of Federal University of Maranhão, Sao Luis, Brazil.

### 2.2. Extraction of Essential Oils

To extract the essential oils, leaves of *A. vulgaris* were dried at 40 °C in an oven with circulating air. These were then sliced into small pieces and subjected to extraction with water by hydro-distillation for 3 h using a Clevenger-type apparatus. The essential oils (1.3 mL) thus obtained was dried over anhydrous sodium sulphate and stored at 4 °C until use.

### 2.3. Identification of Compounds Using Gas Chromatography-Mass Spectrometry (GC-MS)

The analyses of essential oils were performed using GC-2010 plus gas chromatograph (Shimadzu, Japan) coupled to a GCMS-QP2010 SE mass spectrometry detector (Shimadzu, Japan) and equipped with an AOC20i auto-injector (Shimadzu, Japan). A capillary Rtx-5MS column (30 m × 0.25 mm i.d. × 0.25 µm film thickness, Restek, USA) was used for separation. Helium (at a flow rate of 1.0 mL/min) was used as carrier gas. Temperature was kept at 60 °C for 5 min and programmed to reach 240 °C at the rate of 3 °C per min. The samples were injected at the injected temperature of 250 °C. The injection volume was 1.0 μL in 1:30 split ratio. The mass spectra were obtained with electron impact ionization (70 eV) at full scan mode (40 to 500 *m*/*z*), using an ion source at 200 °C.

### 2.4. Identification of Compounds

The compounds were identified by comparing retention indices (RI) and mass spectra with data from the NIST 11.0 MS library and the literature.

### 2.5. Microorganisms and Growth Conditions

*Staphylococcus aureus* ATCC 25923, *Escherichia coli* ATCC 25922, and *Candida albicans* ATCC 90028, already maintained in laboratory, were used for the experiments. The strains were pre-cultured for 24 h on brain heart infusion (BHI; Difco, BD, USA) at 37 °C in the presence of 5% CO_2_.

### 2.6. Determination of Antimicrobial and Anti-Fungal Activities in Essential Oils

The antimicrobial and anti-fungal activities in essential oils of *A. artemisia* were determined through MIC, the microdilution technique according to the broth dilution methodology proposed by the Clinical and Laboratory Standards Institute (CLSI, 2013). In short, sterile 96-well plates were prepared with 50 μL of BHI broth and 50 μL of the essential oil followed by serial dilutions (1:2 to 1:128) done in triplicate After dilution, 2 μL of microbial suspension (0.5 on the MacFarland scale) was added to the samples. Then, 25 μL of chloramphenicol (20 μg/mL) was added to the well as positive control and 50 μL of BHI and 2 μL of microbial suspension were used as growth control. The plate was incubated at 35 °C in an incubator for 24 h for bacteria and 48 h for fungus. After the incubation period, 5 μL of the resazurin (0.1% *w*/*v*) was added.

### 2.7. Parasitological Procedures

Eggs and third stage larvae (L3) were obtained from a donor sheep with a monospecific infection of *Haemonchus contortus*. Experimental procedures were performed in accordance with the guidelines of the Animal Ethics Committee of Maranhão, Federal University and approved under protocol number 23115018061/2011-11.

### 2.8. Egg Hatching Assay (EHA)

Fresh faeces were collected, and the eggs were recovered using 25 μm sieves. Recovered eggs were added to a saturated NaCl solution and centrifuged (3000 rpm) for 3 min; floating eggs were recovered using a 25 μm sieve [40]. A suspension of 100 eggs/well was placed in a plate and 100 µL of essential oils of *A. vulgaris* and control (2%, *v*/*v*, methanol) was added. The essential oils of *A. vulgaris* were diluted in 2% (*v*/*v*) methanol at concentration of 10 mg/mL. Tests were performed with four replicates. The plate was incubated at 27 °C and RH > 80% for 48 h. Larvae and unhatched eggs were counted under an inverted microscope.

### 2.9. Larval Exsheathment Inhibition Assay (LEIA)

This test was performed according to Bahuaud et al. [41]. The essential oil of *A. vulgaris* was diluted in 2% (*v*/*v*) methanol and evaluated at concentration 1.2 mg/mL. The negative control was performed with 2% (*v*/*v*) methanol and PBS (0.1 M phosphate, 0.05 M NaCl, pH 7.2). The L3 larvae were incubated in the different treatments for 3 h at 22 °C. After incubation, the larvae were washed and centrifuged (3000 rpm) three times with PBS. Approximately 1000 larvae/tube was subjected to the artificial exsheathment process by contact with sodium hypochlorite (2.0%, *w*/*v*) and sodium chloride (16.5%, *w*/*v*). Tests were performed with four replicates. The percentages of larval exsheathment process were monitored at 0, 20, 40 and 60 min intervals by observing under an inverted microscope.

### 2.10. Larval Migration Inhibition Assay (LMIA)

The evaluation of larval migration inhibition was performed according to Jackson and Hoste [42]. Initially, *H. contortus* L3 larvae were subjected to the exsheathment process with 2.0% (*w*/*v*) sodium hypochlorite (*w*/*v*). After being sieved, the larvae were centrifuged in distilled water for 5 min at 407× *g* and were re-suspended in distilled water and centrifuged again. Larval suspension (100 μL; approximately 100 larvae) and essential oil of *A. vulgaris* (1000 μL at 10 mg/mL) were added to microtubes. The suspension was incubated for 2 h at 27 °C and ≥80% RH. After that, the tubes were centrifuged at 1500× *g* for 10 min and the supernatant was removed, reducing the volume by approximately 300 μL. Using culture plates, 200 μL of the samples at the concentrations described above were added to the wells, and then an apparatus containing 25 μm sieves were submerged in each well. A 50 μL volume of larval suspension was added to the corresponding apparatus and incubated for 2 h at 27 °C and ≥80% RH. Each apparatus was then removed carefully and the mesh was washed. Lugol was added to each well and larvae of each well and filter were counted. The assay was performed in quadruplicate and methanol (2%, *v*/*v*) was used as a negative control.

## 3. Results and Discussion

### 3.1. Chemical Composition of Essential Oil Extracted from Brazilian A. vulgaris Leaves

The essential oil extracted (0.5%, *v*/*v*) from the leaves of Brazilian *A. vulgaris* was analyzed by GC-MS chromatography (Table 1). The obtained results indicated the presence of 18 chemical compounds in the chromatogram, constituting 100% of the total components detected. The highest compositions of the compounds were 37.45% of caryophyllene, followed by germacrene-D (16.17%) and humulene with 13.66%.

Caryophyllene (β-caryophyllene) [43] is a natural bicyclic sesquiterpene usually found in various essential oils including clove, rosemary, and *Cannabis* [44,45]). This compound is commonly found mixed with α-humulene and isocaryophyllene in essential oils [43]. Caryophyllene is mainly used as a flavoring or fragrance enhancer in spice blends, citrus flavors, chewing gums, soap, detergents, creams or lotions, food products, and beverages. It is known to possess anaesthetic and anti-inflammatory activities [43]. Rasmann and colleagues [46] reported that sesquiterpene olefin (E)-b-caryophyllene plays an essential role in different separate pathways of induced defenses response against herbivores. Caryophyllene can act as antimicrobial agent in defense mechanisms against pathogens [47] such as *Pseudomonas aeruginosa* and *Bacillus subtilis* [48]. Caryophyllene is also reported to have allelopathic potential and shown to inhibit the seedlings development of various plant species [49,50].

Germacrenes, a group of volatile organic hydrocarbon compounds (particularly sesquiterpenes), produced in various plant species are known to act as insecticidal, antimicrobial, and insect pheromones [51,52]. This volatile organic compound has been observed in bryophytes, gymnosperms and angiosperms. Interestingly, germacrene D plays an important role as a precursor in sesquiterpenes synthesis such as selinenes and cadinenes [53,54]. Reportedly, germacrene D as predominant components has been found in the leaves of five plants extracts viz. *Bursera fagaroides*, *B. mirandae*, *B. exselsa*, *B. copallifera*, and *B. ruticola* [55]. Humulene, also known as α- humulene or α-caryophyllene is a naturally occurring monocyclic sesquiterpene characteristic of *Humulus lupulus* [56]. It is also found in *Abies balsamea*, *Salvia officinalis*, *Comptonia peregrine, Cordia verbenacea*, and *Myrica gale* [57,58,59]. Generally terpenes play a role in plants as an anti-herbivore defenses mechanism [60]. Lutz et al. determined the chemical profile of essential oils obtained from seven species of *Artemisia* grown in Canada and studied their antimicrobial and antioxidant activities [61].

Interestingly, monoterpenes hydrocarbons were not found in the essential oil in the present investigation, whereas one diterpene (phytol, 2.94%) was detected. Diterpenes are rare in essential oils and the presence of phytol can be considered as a standard for this species oil. According to Hussein et al. [19], the essential oil from aerial part of mugwort extracted in Egypt contained camphor and 3,5-dimethylcyclohexene as the major components (both with 13.83%), followed by germacrene-D and α-cubenene with 10.44%. However, they found phytol acetate instead of phytol, which were detected in the present work. In contrast to present findings, the monoterpene fraction dominated in the mugwort oils from European countries.

### 3.2. Anti-Microbial Activities Identification

The antimicrobial activity of essential oil of *A. vulgaris* was obtained (Table 2) from microdilution in BHI broth and expressed as Minimum Inhibitory Concentration (MIC), Minimum Bactericidal Concentration (MBC), and Minimum Fungicidal Concentration (MCF). The MIC of essential oil of *A. vulgaris* leaves in *Escherichia coli* ATCC 25922, *Staphylococcus aureus* (ATCC 25923), and *Candida albicans* ATCC 90028 were found at the 1:64, 1:16, and 1:32 dilution titre, respectively. MIC is considered as the lowest concentrations for recognizing the susceptibility of microorganisms to antimicrobial and is applied to judge the performance of all other methods of susceptibility testing. The minimum bactericidal concentration (MBC) affected by extracted essential oil of *A. vulgaris* leaves were reported in *E. coli* at the 1:8 dilution titre. MBC as the lowest concentration act as an antibacterial agent required to kill a microorganism. It could be identified from MIC experiments by sub-culturing to agar plates media that do not contain the test agent. MFC values for *C. albicans* were 1:8 dilution titre. The results revealed variable inhibition activity against *E. coli*, *S. aureus*, and *C. albicans*. The antimicrobial activity was evident with the increase in concentration of essential oil extracted from leaves of *A. vulgaris*. Reportedly, the essential oil of *A. vulgaris* presented lowest bactericidal potential for *E. coli*. The essential oil of *A. vulgaris* showed bactericidal activity for *S. aureus* and fungicide for *C. albicans* in the same concentration [24].

### 3.3. Anthelmintic Activity

The *A. vulgaris* essential oil was tested in high concentrations (10 mg/mL) against eggs (Egg hatching assay) and on two different larval behavior (10 mg/mL and 1.2 mg/mL in LMIA) of main gastrointestinal nematode from small ruminant *H. contortus*. The efficacy of *A. vulgaris* essential oil was low in inhibition of eggs hatch (7.4 ± 6.5%), either in larval exsheathment (4.1 ± 12.8%) and larval migration (1.9 ± 4.1 %). Extracts of several species of *Artemisia* were described as antihelmintic effect against *H. contortus* [62,63,64] and extract of *A. vulgaris* was efficient in vivo models against *Trichinella spiralis* [65]. However, few studies were performed using essential oils of *Artemisia* species against nematodes [66,67]. To our knowledge, this is the first report of *A. vulgaris* essential oil to test against nematode. Besides the efficacy of extracts of *A. vulgaris* on *T. spiralis* [65], the essential oil was not effective against eggs hatch (7.4 ± 6.5%), larval exsheathment (4.1 ± 12.8%), and larval migration (1.9 ± 4.1%). Essential oils of plants with (E)-caryophyllene and β-caryophyllene as main compound showed activity against eggs and adult of *Schistosoma mansoni* and inhibited larval migration of *Strongyloides ratti*, respectively [68,69,70]. β-Caryophyllene was effective for in vitro inhibition of the enzyme Glutathione S-transferase (GST), an important enzyme of detoxification in nematodes [71], however, the data are contradictory, with no efficacy of β-caryophyllene against a root-knot nematode, *Meloidogyne incognita* [72].

## 4. Conclusions

The essential oil from *A. vulgaris* cultivated in Brazil is a potential alternative source of caryophyllene, germacrene D, and humulene. The volatile compounds from this plant species possess antifungal and anti-bacterial properties. Hence, there is a potential for using this essential oil from *A. vulgaris* as disinfectants and preservatives against microorganisms. More studies are required to be focused on isolating the compounds from essential oils and to investigate their biological activities and mode of action in order to use these volatile compounds at commercial level.

## Figures and Tables

**Table 1:** Chemical composition (%) of *Artemisia vulgaris* essential oils.

No.	Compound	RI *	%
1	Borneol	1173	6.80
2	Bornyl acetate	1287	1.46
3	Lavandulyl acetate	1298	2.83
4	Caryophyllene	1420	37.45
5	Humulene	1455	13.66
6	Germacrene D	1482	16.17
7	α-Farnesene	1510	3.11
8	Δ-Cadinene	1524	1.23
9	Epiglobulol	1530	0.58
10	Germacrene B	1558	1.39
11	Nerolidol, acetate	1570	0.49
12	Germacren-d-4-ol	1576	0.93
13	Caryophyllene oxide	1583	5.67
14	Viridiflorol	1592	0.48
15	Isoaromadendrene epoxide	1606	2.17
16	Longipinocarveol, trans-	1634	0.65
17	α-Cadinol	1655	1.99
18	Phytol	2112	2.94

* Retention index.

**Table 2:** Antimicrobial activity of Artemisia vulgaris essential oils against Staphylococcus aureus, Escherichia coli, and Candida albicans *.

-	Microorganism		Positive Control		CC
Microorganism	MIC	MBC/MFC	MIC	MBC/MFC	
*E. coli*	15.6	15.6	0.156 µg/mL	-	+
*S. aureus*	62.4	125	0.078 µg/mL	-	+
*C. albicans*	31.2	125	20 IU/mL	20 IU/mL	+

* Concentration expressed in μL of essential oil/mL BHI broth; C + (Positive Control): For *E. coli* and *S. aureus*, chloramphenicol 0.02 mg/mL; Positive Control for *C. albicans* nystatin 100,000 IU/mL. CC: Positive growth control (there was visible microbial growth). MIC, Minimum Inhibitory Concentration; MBC, Minimum Bactericidal Concentration; MFC, Minimum Fungicidal Concentration.
